# Mechanisms of Diabetic Nephropathy Not Mediated by Hyperglycemia

**DOI:** 10.3390/jcm12216848

**Published:** 2023-10-30

**Authors:** Davide Viggiano

**Affiliations:** Department of Translational Medical Sciences, University of Campania, 80131 Naples, Italy; davide.viggiano@unicampania.it

**Keywords:** fibrosis, diabetic nephropathy, SGLT2i

## Abstract

Diabetes mellitus (DM) is characterized by the appearance of progressive kidney damage, which may progress to end-stage kidney disease. The control of hyperglycemia is usually not sufficient to halt this progression. The kidney damage is quantitatively and qualitatively different in the two forms of diabetes; the typical nodular fibrosis (Kimmelstiel Wilson nodules) appears mostly in type 1 DM, whereas glomerulomegaly is primarily present in type 2 obese DM. An analysis of the different metabolites and hormones in type 1 and type 2 DM and their differential pharmacological treatments might be helpful to advance the hypotheses on the different histopathological patterns of the kidneys and their responses to sodium/glucose transporter type 2 inhibitors (SGLT2i).

## 1. Introduction: Why Hyperglycemia Cannot Explain Everything in Diabetic Nephropathy

Diabetes mellitus (DM) is defined as a chronic hyperglycemic state which is, among the metabolic diseases, not only the most frequent one (422 million people worldwide according to 2023 WHO) but also the state with the most significant amount of literature (946,192 papers on Pubmed, compared to, e.g., 203,674 manuscripts for chronic kidney disease and “only” 174,055 articles for “Atherosclerosis”).

This large amount of data and opinions contrasts with some mysterious aspects of this hyperglycemic state.

The condition of chronic hyperglycemia includes two completely different diseases: type 1 DM (T1DM) is due to a lack of insulin release and occurs in young, thin subjects, whereas the type 2 (T2DM) hyperglycemic state occurs in adult, obese subjects with normal or even elevated insulin release (early T2DM). A third type could be considered the advanced stage of T2DM (advanced T2DM), where endogenous insulin secretion decreases. These three conditions differ in blood composition, the evolution of the disease, and their treatment. Insulin injection is reserved mainly for T1DM and advanced T2DM, whereas T2DM is treated with other hypoglycemic drugs.

Therefore, chronic hyperglycemia can have completely different manifestations and treatments. Organ (brain, kidney, heart, eye) and vascular damage characterize all forms of chronic hyperglycemia, but every type of DM has peculiar aspects.

One puzzling aspect of chronic hyperglycemia is that the vascular and organ damages keep progressing, notwithstanding optimal glycemic control, with some evidence of even an early worsening of vascular injury at the start of the glycemic control [1,2].

This raises a first doubt that hyperglycemia is the only cause of the organ/vascular damage. Maybe we are not able to reach the optimal glycemic control needed to control the disease, or other hormones/metabolites are giving organ damage, or perhaps the DM organism has an internal dynamic that, once started, keeps advancing even in the absence of the starting cause (see, e.g., cancer progression, which does not need the cancerogenic factor to always be present).

When focusing on kidney damage during DM, the paradigm that diabetic nephropathy is initiated and maintained only by hyperglycemia looks inadequate because of the following main criticalities: (i) though chronic hyperglycemia characterizes T1DM and T2DM, the kidney damage is qualitatively and quantitatively different in the two forms [3]; (ii) glycemic control through pharmacological treatment does not halt nephropathy progression [1,4], and insulin infusion does not stop glomerular hyperfiltration initiated by DM [5]; (iii) control of hyperglycemia by insulin is less protective than other hypoglycemic treatments in T2DM; and (iv) the major nephroprotective drugs for diabetic nephropathy are the renin–angiotensin–aldosterone blockers, which do not modify hyperglycemia.

Hyperglycemia, of course, causes enzymatic and non-enzymatic alterations of several proteins [6] and even neurotransmitters [7,8] and must be targeted during the treatment of DM. However, as discussed below, glycemia could also be a marker of the DM state rather than a direct cause of all the problems caused by DM.

Hyperglycemia is certainly directly toxic when very high levels of plasma glucose are reached, a condition termed “hyperosmolar hyperglycemia”, which is an emergency condition causing dehydration [9].

However, in the case of diabetic ketoacidosis (DKA), another emergency condition in uncompensated diabetes, acidosis is not caused by hyperglycemia: it is even possible to develop euglycemic DKA in patients treated with SGLT2 inhibitors [10].

DKA is a clear example of organ damage in DM not caused by hyperglycemia, where high glucose is a biomarker of the disease state (valid for the treatment) rather than of cause of damage [11].

Demonstrations of the direct effect of chronic hyperglycemia (apart from the apparent impact on plasma osmolality) can be obtained, for example, by experimental chronic infusion of glucose in animal models, which induces an 80% decrease in insulin sensitivity already on the first day [12] and an increased number of insulin-releasing cells after just four days of treatment [13]. Furthermore, prolonged infusion of high glucose solutions in the peritoneum, as in peritoneal dialysis, slowly induces a state of fibrosis after many years which is then a significant obstacle for peritoneal dialysis itself [14]. However, since a significant insulin release is present in both cases, it is difficult to disentangle the effects of hyperglycemia from the effects of insulin [15]. In peritoneal dialysis, for example, there is substantial adsorption of glucose and a consequent hyperinsulinism. Using icodextrin as a substitute for glucose in peritoneal dialysis reduces hyperinsulinism [16] and delays (but does not halt) peritoneal fibrosis.

More direct evidence of glucose toxicity can be obtained in vitro by chronically exposing cells to high glucose media without insulin, obviously considering the enormous differences between cells in culture and in vivo conditions. In these conditions, endothelial cells do not die or degenerate: they show more subtle responses such as faster senescences (defined by biochemical parameters) than controls, starting at four weeks and up to 60 days [17]; however, the behavior of cells in culture depends on a huge number of parameters and on the cell line used, and therefore, under other conditions, endothelial cell models in culture may actually respond to high glucose with an enhanced proliferation [18]. Which condition is better for modeling what happens in vivo in humans is, undoubtedly, a hard question. Finally, intensive blood glucose control slows down, but does not halt the T1DM and T2DM nephropathy, though, again, this might in part be interpreted in terms of glucose as a biomarker of disease [19]. These are a few of the large amount of data supporting the paradigm of a direct toxicity of high glucose; in any case, this paradigm does not exclude the toxicity derived from other metabolites (see, e.g., the above example of ketoacidosis).

In summary, if hyperglycemia is the cause of organ and vascular damage, why is its control not sufficient to prevent the injury? Why there is a substantial difference between the different forms of DM? Why hypoglycemic treatments are substantially different in their outcomes? Why organ protection can be obtained by drugs not acting on glycemia?

Even more puzzling is that the hyperglycemic animal models of T1DM (destruction of pancreatic beta cells) do not develop atherosclerotic plaques, whereas T1DM patients do develop atherosclerotic lesions. This problem cannot be dismissed by simply saying that animal models are not good models or that they have different genes: the lack of atherosclerotic lesions in T1DM rodents is totally unexpected because the paradigm of hyperglycemic toxicity is expected to work precisely in the same way in humans and rodents, particularly on the blood vessels, which have the same histological structure and the same blood pressure (120/80 mmHg) in rodents and human beings. If hyperglycemia is the major point, why does it not cause atherosclerosis in rodents? This issue may be pivotal to understanding how to prevent organ damage. Maybe it is a matter of diet (rodents eat mainly fibers), or maybe it is due to the different lipoprotein profiles in rodents (large amounts of HDL). Indeed, only T1DM rodents without a lipoprotein receptor (ApoE knock-out animals) develop atherosclerotic lesions after induction of T1DM [20]. This solid observation, replicated several times in different laboratories, significantly supports the idea that another prominent actor of atherosclerotic lesions in T1DM is dyslipidemia.

Accordingly, the site of formation of atherosclerotic plaques is different in T1DM (non-overweight) vs. T2DM (overweight) patients: they are more distal in T2DM than T1DM [21].

This difference might be consistent with the plasma lipoprotein profile of the two conditions (obesity and dyslipidemia are present in 86–90% of T2DM in contrast with 62% in T1DM [22]) and the hyperinsulinemia of early T2DM.

Furthermore, one should consider that the pancreas beta cells release several other substances apart from insulin, such as C-peptide and amylin. Amylin secretion is reduced in T1DM and normal in T2DM [22]. Therefore, the destruction of these cells in T1DM might be accompanied by insufficient secretion of other hormones apart from insulin, which is not occurring in T2DM. Apart from these, there are other hormonal systems that are interested in T1DM, such as a Glucagon-like peptide 1 [23], or the growth hormone (GH), with the latter being increased in T1DM (with resistance) and decreased in T2DM [24,25].

We shall also consider the off-target effects of insulin, as it is used as a therapy in early T1DM and advanced T2DM: insulin lowers plasma potassium, stimulates growth, and promotes lipid deposition. Insulin is also involved in the formation of foamy cells in atherosclerotic plaques [26].

As a consequence, T1DM is characterized by the reduction of several hormones released by beta cells (e.g., insulin, amylin, C-peptide), whereas T2DM is characterized by glucose-independent metabolic abnormalities (dyslipidemia). In both cases, when insulin treatment is initiated, the off-target effects of the treatment might explain part of the phenomenology of DM.

To summarize, hyperglycemic toxicity is not expected to explain entirely diabetic nephropathy: the lipoprotein (and amino acid, as shown below) profile, the off-target effects of insulin, the absence of hormones produced by beta cells, and the effects of age (discussed below) may come to shape the histological characteristics of the kidney.

Our analysis, therefore, will proceed as follows: we will analyze the differences in diabetic nephropathy in T1 vs. T2DM vs. animal models of DM; these differences shall not be explained by hyperglycemia and could be attributed to non-insulin hormones, hyper-lipidemia, age, or off-target effects of insulin treatment; at variance, similarities in the two forms might be attributed to hyperglycemia.

## 2. T1DM vs. T2DM Nephropathy: Epidemiological Aspects

Over time, DM is accompanied by microvascular changes in many organs, though with different patterns and proportions in T1DM and T2DM. Thus, although the burden of atherosclerotic plaques is similar between the two (65% in T1DM vs. 71% in T2DM) [27], T2DM is characterized by obstructive, non-calcified, and distal plaques in comparison with the calcified, non-obstructive, and proximal plaques of T1DM [21,27].

Accordingly, the kidney is involved with different prevalences in T1DM and T2DM. In a 2015 review, a greater prevalence of diabetic nephropathy in T2DM (about 50% of patients) compared to T1DM (about 33% of patients) has been reported [28]. This has been confirmed in a large population of 17,256 diabetic patients (diagnosed in the years 1965–1990): diabetic nephropathy was present in 44% of T2DM and in 20% of T1DM patients [3]. These percentages do not reflect those of the twenty years before because of a decreased prevalence of diabetic nephropathy in type 1 diabetes due to improved therapies.

The different prevalence of nephropathy in T1DM and T2DM cannot be explained by a different burden of hyperglycemia and therefore might be linked to obesity, hypertension, aging, or perhaps to the hyperinsulinism which characterizes T2DM (Table 1).

## 3. T1DM vs. T2DM Nephropathy: Macroscopic Aspects

It is largely known that the ultrasound feature of kidney nephropathy is an increase in the size of the parenchyma (in terms of volume, maximum diameter, or parenchymal thickness) in the early phase and reduced size in advanced disease. Kidney hypertrophy [36] and kidney atrophy occur with a similar prevalence in T1DM and T2DM.

Indeed, in a cohort of T1DM patients observed on average nine years after the first diagnosis, the kidney was larger (above 99th percentile) in 22% of patients (16% of those without albuminuria, and 44% of those with albuminuria) [37]. At variance, in T2DM on average, the kidney size is also larger than controls in both early (less than 5 years) and advanced DM [39], with a prevalence of about 15% [38].

An analysis at later stages of DM shows that the kidneys are reduced in size (longitudinal diameter less than 10.8cm) in about half of the patients in both T1DM and T2DM, and this correlated with lower eGFR and reduced proteinuria (possibly because of a reduced number of glomeruli) [36]. The reduction in size starts about 10 years after the onset of T2DM [40], whereas in the first 5 years, the kidney size may be greater than normal (14cm on average).

While the reduction in size of the kidneys is shared by all forms of chronic kidney disease [41], the increase in size (kidney hypertrophy) in the early phase is quite peculiar (it is reported only in amyloidosis, renal lymphoma, single kidney or kidney transplant, and obesity). The reason of the increased kidney size is unclear: it would be tempting to hypothesize that it is linked to glomerulomegaly; however, it has been suggested that this is rather due to tubular hypertrophy.

It is important to note that some alterations in kidney weight are acute and reversible.

In animal models of T1DM, the change in kidney weight can occur very rapidly: 10 days after treatment, the kidney is 55% heavier, with a larger size of all structures (glomeruli, tubules), and insulin reduces the kidney weight in only four hours. These modifications could to be linked to hyperglycemia only in acute changes: fasting reduces the kidney weight by 30% in only 18 h, whereas acute hyperglycemia increases the kidney weight by 22% in the same time [42]. These acute changes are very unlikely to mirror the kidney hypertrophy in humans, which takes years to occur.

These acute changes might be interpreted in terms of a shift/retention of fluids. They are very unlikely to mirror the kidney hypertrophy in humans, which takes years to occur. Therefore, we are rather interested in changes that need years to appear and are stable in time.

In uninephrectomized animals, the remaining kidney has a larger size and hyperfiltration. However, the reduction of filtration induced by empagliflozin induces an even larger kidney size, suggesting that the kidney dimension does not depend on glomerulomegaly but on tubular hypertrophy [43]. Furthermore, SGLT2 knockout mice also show tubular hypertrophy with increased kidney size without alteration in GFR, and the induction of diabetes in these animals further increases the kidney size without greatly modifying the GFR [44]. Interestingly, SGLT2 inhibitors also prevent retinal microangiopathy [45].

Studies in animal models fed with a fat diet and with the absence of an insulin receptor strongly suggest that insulin is by itself responsible for the kidney hypertrophy [45]. Thus is tempting to attribute the larger kidney size in T2DM to the hyperinsulinism or to obesity, because kidney weight increases according to the body mass index [40,46]. Furthermore, obesity is accompanied by glomerulomegaly and tubular hypertrophy, in the absence of diabetes [47].

In summary, kidney atrophy has a similar prevalence and interpretation in both T1DM and T2DM, that is a reduction in the number of nephrons, as in all other chronic kidney diseases.

In contrast, kidney hypertrophy is observed in a minority of cases of T1DM and T2DM, suggesting that it might be linked to hyperglycemia, but this is not sufficient to cause the kidney enlargement. Its similarity to that of obesity suggests that it is linked to dyslipidemia (Table 1). However, the increased kidney size in the case of single kidney and altered function of proximal tubules (in absence of dyslipidemia/hyperglycemia) may suggest that a hormonal stimulus can induce kidney growth in all these conditions.

## 4. T1DM vs. T2DM Nephropathy: Microscopic Aspects

In contrast with kidney hypertrophy, nephropathy is quantitatively and qualitatively different between T1DM and T2DM.

T1DM nephropathy is always characterized by damage to the glomerulus, whereas in T2DM, nephropathy minimal glomerular lesions are present when proteinuria appears [48]. This conclusion derives from comparisons of separate studies and no study has systematically compared the kidney histology in the two conditions. According to a 2020 review, in T1DM, glomerular lesions start 2 years after the onset of the disease, with the thickening of glomerular basal membranes, followed by mesangial expansion, collagen IV deposition and formation of Kimmelstiel Wilson nodules, arteriolar hyalinosis, and tubular obstruction with atubular glomeruli [49,50,51,52,53]. The glomerular volume is not increased [54].

At variance, in T2DM with proteinuria, diabetic glomerulopathy is present in 30–50% of cases. In the other cases, no glomerular lesions can be detected in 10% of cases whereas the remaining patients show prominent tubulointerstitial damage, arteriolar hyalinosis atherosclerosis [55,56]. In the case of glomerulopathy, this is always accompanied by diabetic retinopathy, whereas the latter is present in only 50% of cases without glomerulopathy. The glomerular volume is increased [35].

Several differences between T1DM and T2DM can explain the histological differences. T2DM is usually accompanied by older age, obesity, and hypertension (up to 90% of cases, compared to 14% of cases in T1DM [30,57]). The hypertension in T2DM is mostly systolic, whereas the one in T1DM is diastolic; consistently, the cause of death in T1DM was end-stage kidney disease, whereas cardiovascular disease dominates in T2DM [29]. Another intriguing difference, though, pertains to medications: T2DM is not treated with insulin at first, whereas this is the first choice in T1DM. This raises the possibility that part of the lesion is caused by insulin itself.

Glomerulomegaly is more evident in T2DM than T1DM: on average, glomeruli are larger in T2DM, with a greater mesangio-urinary surface, capillary length, and filtration surface, without differences in the proportion of mesangial or matrix material or width of the glomerular basement membrane [35].

It is important to note that the glomerulomegaly in T2DM could derive from obesity, which often accompanies this hyperglycemic state: the glomerular volume increases as a function of body mass index [58] or body surface area [59], even in the presence of another glomerulopathy (e.g., IgA nephropathy) [60]. At variance, proximal tubule hypertrophy seems to be related to proteinuria [47,61].

## 5. Glomerulomegaly versus Hyperfiltration

Glomerulomegaly is an increase in the size of the glomeruli, and it often accompanies T2DM and less often T1DM. It is an anatomical modification that requires an increase in the total number of cells. Usually, it is also accompanied by an increased filtration rate, which derives from the larger filtration surface of the glomeruli. Glomerulomegaly is observed in only a few conditions such as diabetes mellitus, single kidney, obesity, a low glomerular number at birth, and glomerulosclerosis [62].

At variance, hyperfiltration is a functional term. It refers to an increase in the glomerular filtration rate and, usually, to a lower-than-expected level of plasma creatinine. Hyperfiltration may derive from an anatomical enlargement of the glomerular filtration surface as in diabetic glomerulomegaly, but more often, it derives from a change in the plasma flow in the glomerulus: every day we have transient states of hyperfiltration after a meat meal [63,64], which are not due to anatomical changes. Functional hyperfiltration occurs after the infusion of amino acids, during sport, and during pregnancy. In DM, hyperfiltration has been observed in both T1DM and T2DM, with an increase in GFR in the range of 16–27% in both types of DM [22].

Some information about glomerulomegaly derives from observations on patients with a single kidney. This enlarges by a great amount and this seems to be driven by a greater flow of amino acids in the kidney and subsequent activation of mTOR [65].

Furthermore, a deletion of a protein in the proximal tubule, Pten, also induces glomerulomegaly, because of the activation of mTOR [65]. Consistently, rapamycin, an mTOR inhibitor, reduced glomerulomegaly.

## 6. T1DM vs. T2DM: Basement Membrane and the Kimmelstiel Wilson Nodules

Among the earliest histological/structural alterations that can be observed in DM are the increased intima-media thickness, an altered vascular bed in the retina, and nodular and interstitial fibrosis in the glomeruli (Kimmelstiel Wilson nodules).

Kimmelstiel Wilson nodules are histological lesions that involve the mesangium and its interface with endothelial cells, that is the basement membrane and extracellular matrix. The presence of inter-capillary connective tissue was first noted in 1936 by the German pathologist/biochemist Paul Kimmelstiel and the British nephrologist Clifford Wilson at Harvard. They were trying to differentiate intra- and extra-capillary glomerulonephritis from an “intercapillary glomerulosclerosis”, a hyaline lesion that resembled an amyloid or aging kidney. They observed that the lesion was present in diabetic cases, though “only a small proportion of cases of diabetes appears to show this lesion” [66]. The nodules are characterized by nodular sclerosis (deposition of collagen in spherical or ovoidal, “onion-like” structures in one or more glomerular lobules), mesangial sclerosis, arteriolar hyalinosis, dilatation of capillaries (microaneurysms), exudation, and thickening of the glomerular basement membrane. Animal models of T1DM (insulin deficiency after streptozotocin treatment) do not develop these nodules. Early studies suggested that these lesions are not linked to obesity [67].

Several mechanisms have been proposed to explain the nodular deposition of collagen in lobules of the glomeruli. Early work suggested an origin different from mesangial sclerosis [67], and that focal mesangiolysis [66] and the subsequent mesangial repair might be an early feature of the lesions [68]. Considering the timing of histopathologic lesions, the thickening of the basement membrane seems to occur first, followed by mesangial expansion and arteriolar hyalinization; mesangiolysis follows with increased mesangial matrix, and podocyte loss [68]. T1DM has the peculiarity to have sclerosis at the junction of glomeruli and proximal tubule, leading to atubular glomeruli [68].

The presence of Kimmelstiel Wilson nodules often is paralleled by several other diabetes complications such as proliferative retinopathy [67]. In a large study on 256 patients, Kimmelstiel Wilson nodules were associated with younger age, diabetic retinopathy, more severe proteinuria and renal insufficiency, microaneurysms, tubular atrophy/interstitial fibrosis, arteriosclerosis and arteriolar hyalinosis, and increased IgG/IgA; conversely, no association was found with obesity, hypertension, or the amount of HbA1c [68]. The latter would exclude that uncontrolled hyperglycemia causes these nodules. The association between Kimmelstiel Wilson nodules and peripheral neuropathy has not been analyzed systematically, though a strong correlation is known to exist between diabetic nephropathy and neuropathy [68].

The molecular mechanism of nodular glomerulosclerosis has been difficult to understand because classical models of DM do not show this alteration. However, recently, the lesion has been produced in mice with T1DM by overexpressing VEGF-A in the podocytes [69]. Podocytes are the main source of VEGF-A in the glomerulus. Alternatively, T1DM mice can produce nodular glomerulosclerosis if eNOS is deleted, thereby hindering the production of NO in endothelial cells [70]. Finally, mice overexpressing VEGF-A in the podocytes and lacking eNOS also produce nodular glomerulosclerosis [66], in the absence of hyperglycemia, diabetic milieu, or hypertension. This experimental model has suggested that mesangiolysis coexists with glomerulosclerosis, the latter being caused by VEGF-A and NO insufficiency. At present, the mechanism by which insulin, C-peptide, hyperglycemia, dyslipidemia, or aging are altering the VEGF/NO balance in glomeruli is unknown.

One possible common feature among these three structural alterations is the increased basement membrane thickness in all these districts.

The basement membrane can be considered a condensation of the connective tissue adjacent to an epithelium such as the endothelium. It is composed of collagen type VII, type III and type IV, fibrillin, perlecan, laminin, nidogen, integrins, entactins, and dystroglycans [71]. The basement membrane anchors the epithelium to the connective tissue: any alteration will cause detachment of the epithelium with formation of atheroma (in blood vessels), tubular atrophy, proteinuria, deposition of collagen under the capillaries (Kimmelstiel Wilson nodules), and several other phenomena typical of diabetes. Furthermore, it is needed for endothelial cell differentiation and its damage causes endothelium de-differentiation. It also prevents the spread of malignant epithelial cells, and hence, its damage may increase metastasis [72].

Though Kimmelstiel Wilson nodules were first observed in post-mortem kidneys, the advent of kidney biopsies has also confirmed their relevance in living subjects [73]. New imaging methods on kidney biopsies, such as infrared spectroscopy, promise to give detail about the composition of nodules [68]. Unfortunately, no techniques are available to identify the nodules in vivo as their size is below the resolution of current imaging methods.

Proteome analysis of the basement membrane of arteries in T2DM shows an enrichment of typical proteins in arteries [71,74], that is a basement membrane accumulation in both small and large vessels.

Consistently, proteome analysis of glomeruli from diabetic patients has shown an increase in several proteins of the basement membrane including Collagen Type VI, Collagen, Type IV, Laminin, Fibrillin, perlecan, nidogen, and nephronectin, with the latter mediating mesangial adhesion [75].

Proteomic analysis of the Kimmelstiel Wilson nodules in T2DM has shown accumulation of collagen IV, but no change in fibrinogen, fibronectin, perlecan, or laminin [76]. Furthermore, these nodules contain proteins that do not pertain to basement membranes: complement C9 and apolipoprotein E [76]. This intriguing result suggests that these nodules have a substantially different composition from basement membranes.

Since these nodules are more often seen in T1DM than type 2, it is unlikely that hyperglycemia is the only cause of their formation (see Table 1).

## 7. The Non-Glycemic Effects of Insulin Therapy

Apart from the largely known effect on glycemia, insulin has complex effects on several other metabolites, electrolytes, and an intrinsic impact on tissue growth which may be interesting when considering organ hypertrophy and vascular occlusion in DM.

Insulin has two primary receptors named IR-A and IR-B originating by alternate splicing of a single gene, INSR. IR-B is ubiquitous and responsible for the regulation of glucose, fat, and proteins in response to insulin. IR-B is also activated by IGF-I and IGF-II.

In obese subjects, tissues become less responsive to insulin because of a reduced number of receptors or their biochemical modifications. However, not all organs and tissues become insulin resistant at the same time and the term “tissue-specific insulin resistance” is therefore used. Usually liver and adipose tissue are affected first by insulin resistance, whereas the muscle develops resistance at a slower rate [77,78,79]. Even the brain might be affected by insulin resistance [80,81]. This also means that insulin treatment has different effects on different organs and some organs might be over-stimulated by an insulin treatment that is titrated only on glycemia.

The insulin receptor is also expressed in the kidney, mostly in the tubules (70% of positive cells) but also in the glomeruli (20% of positive cells) and fibroblasts (5% of positive cells; data from The Human Protein Atlas database). Notwithstanding this expression, insulin treatment does not change kidney hemodynamics and GFR in normal subjects [82]. However, in T2DM, insulin increases the albumin excretion and causes hyperuricemia [83,84], and this effect persists when tissue-specific insulin resistance affects other organs [82]. This provides strong support to the idea that some of the phenomena observed in the kidney of T2DM is caused by insulin itself rather than by hyperglycemia.

A metabolome analysis of T2DM patients treated with insulin has shown an increase in plasma amino acids (proline, glycine, serine, threonine, methionine, pyroglutamic acid, glutamine, and lysine) [34]. By contrast, metformin treatment led to a different signature with elevated levels of leucine/isoleucine and reduced carnitine, tyrosine, and valine [85].

At variance, in T1DM insulin treatment caused the reduction of acetate, allantoin, ketones, plasma amino acids, and amino acid metabolites [86].

Though a systematic analysis of insulin effects on metabolome in T1DM and T2DM is unavailable, these data confirm the numerous metabolic effects of insulin with possible differential phenomena in T1 and T2DM.

Insulin is also responsible for tissue architecture changes.

1. Insulin promotes subcutaneous fat deposition, which has been demonstrated in both animal models [87] and patients [88]. The consequent increase in total body weight is an adverse effect of insulin treatment [89]. Insulin is also involved in the formation of foamy cells in atherosclerotic plaques [26]. Consistently, hyperinsulinaemia can result in adiposity in the human fetus.
2. Insulin promotes growth in connective or musculoskeletal tissues [90]. Consistently, hyperinsulinemia (diabetic mother) can result in increased body size of the fetus. Insulin can also stimulate fibroblasts in vitro [91], inducing myocardial fibrosis in animal models in vivo [92]. Insulin can also stimulate cancer cell growth, such as human colon cancer cells [93]. Finally, insulin may have proangiogenic effects on endothelial cells [94], though with some effect on faster senescence as discussed above. This effect may explain the slower wound healing in T1DM, which is not present in T2DM [95]. Therefore, at least, the latter phenomenon is not linked to hyperglycemia toxicity.

## 8. Hyperinsulinism and Nephropathy

In T2DM treated with exogenous insulin, the endogenous insulin levels, indexed by C-peptide levels, decrease over the years [33]. Notably, in these exogenous-insulin treated patients, diabetic nephropathy is accompanied by higher levels of fasting C-peptide [33]. Similarly, in a study on 585 T2DM patients without exogenous insulin, the greater baseline insulin levels and C-peptide are correlated to the development of retinal disease [96]. This leads to the hypothesis that diabetic nephropathy emerges when insulin secretion is greater than normal.

Premature insulin treatment in T2DM leads to weight gain [97] and a high C-peptide concentration (indexing endogenous insulin release) may be related to microvascular damage [31], chemiotaxis of inflammatory cells and smooth muscle cells, and proliferation thus contributing to atherosclerosis [98]. A recent metanalysis suggested a link between atherosclerosis and high C-peptide levels [99].

A more indirect indication of insulin concentration in T1DM and T2DM is the non-glycemic effects of insulin: potassium, lipid accumulation, and glycogen stores. Unfortunately, a systematic analysis of these effects is lacking. We know that potassium may be more elevated in T2DM than controls in a population of patients with angina [100]. However, in this study, the patients with greater potassium already had a significant reduction of glomerular filtration rate and no information is available on C-peptide levels.

In summary, it is tempting to speculate that glomerulomegaly in T2DM is not due to hyperglycemia but to increased insulin levels. Conversely, the absence of C-peptide replacement in T1DM might be linked to other phenomena.

Indeed, the addition of C-peptide to insulin infusion has some beneficial effects in T1DM [101,102,103]. Furthermore, the short-term infusion of supraphysiological doses of human C-peptide to diabetic rats resulted in the normalization of the glomerular filtration rate (GFR).

In agreement with this hypothesis, a study in rodent models showed that the infusion of C-peptide was able to decrease an acute form of glomerulomegaly caused by pancreatic beta cells destruction [104]. In this case, no insulin treatment was initiated in rats and glomerulomegaly was observed after only 14 days from the onset of disease and albuminuria, starting as early as 4 weeks after the onset. This suggests that the acute glomerulomegaly was not linked to hyperglycemia (the C-peptide had no effects on glycemia).

## 9. Why the Diabetic Nephropathy Occurs at a Faster Pace in Mice or Rats or Cell Cultures? Role of Aging

The analysis of results from animal models and cell cultures should take into account the chronic manifestations of any treatment: indeed, as reported above, when considering organ weights, these can be modified in as short a time frame as a few hours of hyperglycemia. These acute changes have probably little if nothing to do with the chronic changes observed in humans, which occur in a time-frame of years and require the change in composition of basement membranes and cellular proliferation.

However, in animal models of DM, chronic changes appear after weeks or months, faster than in human beings. There are various possible explanations. Maybe, we are able to detect earlier changes in animals than in humans. Or maybe, the damage induced in animals is much worse than that in humans.

It is instructive to compare this with animal models of other chronic, slow diseases. For example, animal models of Alzheimer do not develop a reasonable brain alteration when the human genes are inserted: one must induce a dramatic genetic change to obtain a model that will produce brain damages in a few months (that is much faster than in humans).

Similarly, the ob/ob mice, a widely used model of obesity, indeed have a reduction in their lifespan, as in humans, which means that the disease occurs at a faster rate in this model [105]. However, these animals mimic a very rare, genetically determined and extremely severe form of obesity in humans: they weigh about three times more than control animals, which would correspond to a human being of 270 kg (or more, if one considers the ratio body weight/body surface).

In experimental animals treated with streptozotocin (to destroy pancreatic beta cells) and insulin, fibronectin deposition in the kidney occurs already after only 4 weeks, but the kidney size remained the same [106]. When compared to human subjects, the same study [3] confirms that in both T1DM and T2DM patients the nephropathy starts appearing no less than 4 years after the beginning of the disease. Clearly, small rodents appear to have a more rapid onset of the damage once the insulin deficit appears.

When considering in vitro systems, endothelial cells in culture show a response to hyperglycemia after the same period as in small rodents: 4 weeks [17].

One simple hypothesis of the faster appearance of diabetic lesions in small rodents and cell cultures is that mice/cells age at a faster rate. If this hypothesis is true, that is that diabetic lesions require some aspects of aging, one should see less lesions in children.

This is true: While Kimmelstiel Wilson nodules are primarily associated with adults, being reported rarely in single cases of children and adolescents with diabetes (see, e.g., [107]). These cases usually also present with hypercholesterolemia. Interestingly, one pediatric case with Kimmelstiel Wilson nodules did not suffer from DM but from cystic fibrosis [108].

A second consequence would be that younger patients with T1DM should have a reduced per year risk of undergoing hemodialysis. Indeed, a study involving a large cohort of T1DM shows that ESRD was lowest in patients whose diagnosis occurred at younger than 5 years: the prevalence of hemodialysis is less than 3% in 9-year-old children, and 4% in children who are 14 years or older [109]. The same applies in mice: older mice or rats treated with STZ exhibit more extensive kidney lesions than younger animals [110].

## 10. Conclusions: A Working Hypothesis

The overall picture derived from the analysis of differences between T1DM and T2DM, the non-hyperglycemic damages in DM and the structural/biochemical alterations in DM allows for proposing the following phenomena of diabetic nephropathy not mediated by hyperglycemia:

1. Aging and dyslipidemia are relevant factors for the subsequent alterations; decreased C-peptide in T1DM, hyperinsulinism, and hyper -aminoacidemia in T2DM contribute to the different patterns of lesions in the hyperglycemic states.
2. Insulin induces an increased connective tissue growth and tubular hypertrophy.
3. Tubular hypertrophy induces an increased kidney size.
4. The connective tissue immediately adjacent to the endothelia, that is the basement membrane, is altered, contributing to nodules in the glomeruli.
5. Greater plasma amino acid contents induced by insulin and mTOR activation induce hyperfiltration and glomerulomegaly.

## Figures and Tables

**Table 1:** Similarities and differences of nephropathy among hyperglycemic conditions cited in the text.

Characteristic	T1DM	T2DM
Obesity [22]	62%	86–90%
Age	Young	Old
Cause of death [29]	End stage kidney disease	Cardiovascular events
Hypertension [29,30]	14% (diastolic)	32–90% (systolic)
Plasma profile		
Hyperglycemia	Yes	Yes
Dyslipidemia [22]	Yes	Yes
C-peptide [31]	Low	Normal or high
Amylin [32]	Low	Normal
Insulin treatment	Yes	No (only in late stages)
Endogenous Insulin [33]	Low	Normal or high (Low in advanced stage)
Aminoacids [34]	Decreased	Increased
Growth Hormone (GH) [24,25]	Increased	Decreased
Kidney Morphological changes		
Tubular hypertrophy	Yes (Data from animal models)	Yes (Data from animal models)
Basement membrane thickening [35]	Frequent (Data from animal models)	Less frequent (Data from animal models)
Atherosclerotic plaques [21]	Calcified, non-obstructive and proximal	Obstructive, non-calcified and distal
Glomerulomegaly [35]	Less frequent	Frequent
Glomerulopathy	100%	50%
Kimmelstiel Wilson nodules	Frequent	Rare
Kidney hypertrophy [36,37,38]	22%	15%
Kidney Functional changes		
Proteinuria	Yes	Yes
Hyperfiltration [22]	Yes (10–67%)	Yes (6–73%)
Diabetic nephropathy (proteinuria, reduced eGFR) [28]	20–33%	44–50%

## Data Availability

Not applicable.

## References

[1] Perkins B.A. (2022). The uncomfortable truth about kidney disease in type 1 diabetes. Lancet Diabetes Endocrinol..

[2] Bain S.C., Klufas M.A., Ho A., Matthews D.R. (2019). Worsening of diabetic retinopathy with rapid improvement in systemic glucose control: A review. Diabetes Obes. Metab..

[3] Yokoyama H., Okudaira M., Otani T., Sato A., Miura J., Takaike H., Yamada H., Muto K., Uchigata Y., Ohashi Y. (2000). Higher incidence of diabetic nephropathy in type 2 than in type 1 diabetes in early-onset diabetes in Japan. Kidney Int..

[4] Sattar N., Wannamethee S.G., Forouhi N.G. (2008). Novel biochemical risk factors for type 2 diabetes: Pathogenic insights or prediction possibilities?. Diabetologia.

[5] Christiansen J.S., Frandsen M., Parving H.H. (1981). The effect of intravenous insulin infusion on kidney function in insulin-dependent diabetes mellitus. Diabetologia.

[6] Copeland K.R., Yatscoff R.W., Thliveris J.A., Mehta A., Penner B. (1987). Non-enzymatic glycation and altered renal structure and function in the diabetic rat. Kidney Int..

[7] Ruocco L., Viggiano D., Pignatelli M., Iannaccone T., Rimoli M.G., Melisi D., Curcio A., De Lucia S., Carboni E., Gironi-Carnevale U.A. (2008). Galactosilated dopamine increases attention without reducing activity in C57BL/6 mice. Behav. Brain Res..

[8] Ruocco L.A., Viggiano D., Viggiano A., Abignente E., Rimoli M.G., Melisi D., Curcio A., Nieddu M., Boatto G., Carboni E. (2008). Galactosylated dopamine enters into the brain, blocks the mesocorticolimbic system and modulates activity and scanning time in Naples high excitability rats. Neuroscience.

[9] Rosenbloom A.L. (2007). Hyperglycemic crises and their complications in children. J. Pediatr. Endocrinol. Metab..

[10] Long B., Lentz S., Koyfman A., Gottlieb M. (2021). Euglycemic diabetic ketoacidosis: Etiologies, evaluation, and management. Am. J. Emerg. Med..

[11] Modi A., Agrawal A., Morgan F. (2017). Euglycemic Diabetic Ketoacidosis: A Review. Curr. Diabetes Rev..

[12] Topp B.G., McArthur M.D., Finegood D.T. (2004). Metabolic adaptations to chronic glucose infusion in rats. Diabetologia.

[13] Alonso L.C., Yokoe T., Zhang P., Scott D.K., Kim S.K., O’Donnell C.P., Garcia-Ocaña A. (2007). Glucose infusion in mice: A new model to induce beta-cell replication. Diabetes.

[14] Shirai Y., Miura K., Ike T., Sasaki K., Ishizuka K., Horita S., Taneda S., Hirano D., Honda K., Yamaguchi Y. (2022). Cumulative Dialytic Glucose Exposure is a Risk Factor for Peritoneal Fibrosis and Angiogenesis in Pediatric Patients Undergoing Peritoneal Dialysis Using Neutral-pH Fluids. Kidney Int. Rep..

[15] Brands M. (2000). Chronic intravenous glucose infusion causes moderate hypertension in rats. Am. J. Hypertens..

[16] Amici G., Orrasch M., Da Rin G., Bocci C. (2001). Hyperinsulinism reduction associated with icodextrin treatment in continuous ambulatory peritoneal dialysis patients. Adv. Perit. Dial..

[17] Bertelli P.M., Pedrini E., Hughes D., McDonnell S., Pathak V., Peixoto E., Guduric-Fuchs J., Stitt A.W., Medina R.J. (2022). Long term high glucose exposure induces premature senescence in retinal endothelial cells. Front. Physiol..

[18] Li X., Liu Y., Li Y., Xie N., Yan Y., Chi Y., Zhou L., Xie S., Wang P. (2016). High glucose concentration induces endothelial cell proliferation by regulating cyclin-D2-related miR-98. J. Cell. Mol. Med..

[19] Alicic R.Z., Rooney M.T., Tuttle K.R. (2017). Diabetic Kidney Disease: Challenges, Progress, and Possibilities. Clin. J. Am. Soc. Nephrol..

[20] Choi J.S.Y., de Haan J.B., Sharma A. (2022). Animal models of diabetes-associated vascular diseases: An update on available models and experimental analysis. Br. J. Pharmacol..

[21] Burke A.P., Kolodgie F.D., Zieske A., Fowler D.R., Weber D.K., Varghese P.J., Farb A., Virmani R. (2004). Morphologic Findings of Coronary Atherosclerotic Plaques in Diabetics. Arterioscler. Thromb. Vasc. Biol..

[22] Tonneijck L., Muskiet M.H.A., Smits M.M., van Bommel E.J., Heerspink H.J.L., van Raalte D.H., Joles J.A. (2017). Glomerular Hyperfiltration in Diabetes: Mechanisms, Clinical Significance, and Treatment. J. Am. Soc. Nephrol..

[23] Salvatore T., Carbonara O., Cozzolino D., Torella R., Sasso F.C. (2007). Adapting the GLP-1-signaling system to the treatment of type 2 diabetes. Curr. Diabetes Rev..

[24] Koren D. (2022). Growth and development in type 1 diabetes. Curr. Opin. Endocrinol. Diabetes. Obes..

[25] Giustina A., Wehrenberg W.B. (1994). Growth hormone neuroregulation in diabetes mellitus. Trends Endocrinol. Metab..

[26] Park Y.M., Kashyap S.R., Major J.A., Silverstein R.L. (2012). Insulin promotes macrophage foam cell formation: Potential implications in diabetes-related atherosclerosis. Lab. Investig..

[27] Djaberi R., Schuijf J.D., Boersma E., Kroft L.J.M., Pereira A.M., Romijn J.A., Scholte A.J., Jukema J.W., Bax J.J. (2009). Differences in atherosclerotic plaque burden and morphology between type 1 and 2 diabetes as assessed by multislice computed tomography. Diabetes Care.

[28] Thomas M.C., Brownlee M., Susztak K., Sharma K., Jandeleit-Dahm K.A.M., Zoungas S., Rossing P., Groop P.-H., Cooper M.E. (2015). Diabetic kidney disease. Nat. Rev. Dis. Prim..

[29] Parving H.H. (2001). Diabetic nephropathy: Prevention and treatment. Kidney Int..

[30] Haile T.G., Mariye T., Tadesse D.B., Gebremeskel G.G., Asefa G.G., Getachew T. (2023). Prevalence of hypertension among type 2 diabetes mellitus patients in Ethiopia: A systematic review and meta-analysis. Int. Health.

[31] Alves M.T., Ortiz M.M.O., dos Reis G.V.O.P., Dusse L.M.S., das Graças Carvalho M., Fernandes A.P., Gomes K.B. (2019). The dual effect of C-peptide on cellular activation and atherosclerosis: Protective or not?. Diabetes. Metab. Res. Rev..

[32] Hieronymus L., Griffin S. (2015). Role of Amylin in Type 1 and Type 2 Diabetes. Diabetes Educ..

[33] Dario T., Riccardo G., Silvia P., Mikiko W., Daria M., Andrea P., Giuseppe D., Elvira F., Paolo P., Silvia M. (2021). The utility of assessing C-peptide in patients with insulin-treated type 2 diabetes: A cross-sectional study. Acta Diabetol..

[34] Taya N., Katakami N., Omori K., Arakawa S., Hosoe S., Watanabe H., Takahara M., Miyashita K., Nishizawa H., Matsuoka T.-A. (2021). Evaluation of change in metabolome caused by comprehensive diabetes treatment: A prospective observational study of diabetes inpatients with gas chromatography/mass spectrometry-based non-target metabolomic analysis. J. Diabetes Investig..

[35] White K.E., Marshall S.M., Bilous R.W. (2007). Are glomerular volume differences between type 1 and type 2 diabetic patients pathologically significant?. Diabetologia.

[36] Rigalleau V., Garcia M., Lasseur C., Laurent F., Montaudon M., Raffaitin C., Barthe N., Beauvieux M.-C., Vendrely B., Chauveau P. (2010). Large kidneys predict poor renal outcome in subjects with diabetes and chronic kidney disease. BMC Nephrol..

[37] Zerbini G., Bonfanti R., Meschi F., Bognetti E., Paesano P.L., Gianolli L., Querques M., Maestroni A., Calori G., Del Maschio A. (2006). Persistent renal hypertrophy and faster decline of glomerular filtration rate precede the development of microalbuminuria in type 1 diabetes. Diabetes.

[38] Ham Y.R., Lee E.J., Kim H.R., Jeon J.W., Na K.R., Lee K.W., Choi D.E. (2023). Ultrasound Renal Score to Predict the Renal Disease Prognosis in Patients with Diabetic Kidney Disease: An Investigative Study. Diagnostics.

[39] Wirta O.R., Pasternack A.I. (1995). Glomerular filtration rate and kidney size in type 2 (non-insulin-dependent) diabetes mellitus. Clin. Nephrol..

[40] Omer M.A.A., Eljack A.H., Gar-alnabi M.E.M., Mahmoud M.Z., Elseid M., Edam G.A. (2014). Ultrasonographic Characteristics of Diabetes Impacts in Kidneys’ Morphology. Open J. Radiol..

[41] Romano G., Mioni R., Danieli N., Bertoni M., Croatto E., Merla L., Alcaro L., Pedduzza A., Metcalf X., Rigamonti A. (2022). Elevated Intrarenal Resistive Index Predicted Faster Renal Function Decline and Long-Term Mortality in Non-Proteinuric Chronic Kidney Disease. J. Clin. Med..

[42] Rasch R., Dørup J. (1997). Quantitative morphology of the rat kidney during diabetes mellitus and insulin treatment. Diabetologia.

[43] Tauber P., Sinha F., Berger R.S., Gronwald W., Dettmer K., Kuhn M., Trum M., Maier L.S., Wagner S., Schweda F. (2021). Empagliflozin Reduces Renal Hyperfiltration in Response to Uninephrectomy, but Is Not Nephroprotective in UNx/DOCA/Salt Mouse Models. Front. Pharmacol..

[44] Vallon V., Rose M., Gerasimova M., Satriano J., Platt K.A., Koepsell H., Cunard R., Sharma K., Thomson S.C., Rieg T. (2013). Knockout of Na-glucose transporter SGLT2 attenuates hyperglycemia and glomerular hyperfiltration but not kidney growth or injury in diabetes mellitus. Am. J. Physiol. Physiol..

[45] Sabaner M.C., Duman R., Dogan M., Akdogan M., Vurmaz A., Bozkurt E., Beysel S. (2021). Do SGLT2 inhibitors prevent preclinical diabetic retinopathy? A Prospective Pilot Optical Coherence Tomography Angiography Study. J. Fr. Ophtalmol..

[46] Mandal R., Loeffler A.G., Salamat S., Fritsch M.K. (2012). Organ weight changes associated with body mass index determined from a medical autopsy population. Am. J. Forensic Med. Pathol..

[47] Tobar A., Ori Y., Benchetrit S., Milo G., Herman-Edelstein M., Zingerman B., Lev N., Gafter U., Chagnac A. (2013). Proximal tubular hypertrophy and enlarged glomerular and proximal tubular urinary space in obese subjects with proteinuria. PLoS ONE.

[48] Di Vincenzo A., Bettini S., Russo L., Mazzocut S., Mauer M., Fioretto P. (2020). Renal structure in type 2 diabetes: Facts and misconceptions. J. Nephrol..

[49] Osterby R. (1972). Morphometric studies of the peripheral glomerular basement membrane in early juvenile diabetes. I. Development of initial basement membrane thickening. Diabetologia.

[50] Najafian B., Crosson J.T., Kim Y., Mauer M. (2006). Glomerulotubular junction abnormalities are associated with proteinuria in type 1 diabetes. J. Am. Soc. Nephrol..

[51] Harris R.D., Steffes M.W., Bilous R.W., Sutherland D.E., Mauer S.M. (1991). Global glomerular sclerosis and glomerular arteriolar hyalinosis in insulin dependent diabetes. Kidney Int..

[52] Mauer M., Caramori M.L., Fioretto P., Najafian B. (2015). Glomerular structural-functional relationship models of diabetic nephropathy are robust in type 1 diabetic patients. Nephrol. Dial. Transplant.

[53] Mauer S.M., Steffes M.W., Ellis E.N., Sutherland D.E., Brown D.M., Goetz F.C. (1984). Structural-functional relationships in diabetic nephropathy. J. Clin. Investig..

[54] Steffes M.W., Schmidt D., Mccrery R., Basgen J.M. (2001). Glomerular cell number in normal subjects and in type 1 diabetic patients. Kidney Int..

[55] Moriya T., Suzuki Y., Inomata S., Iwano M., Kanauchi M., Haneda M. (2014). Renal histological heterogeneity and functional progress in normoalbuminuric and microalbuminuric Japanese patients with type 2 diabetes. BMJ Open Diabetes Res. Care.

[56] Fioretto P., Mauer M., Brocco E., Velussi M., Frigato F., Muollo B., Sambataro M., Abaterusso C., Baggio B., Crepaldi G. (1996). Patterns of renal injury in NIDDM patients with microalbuminuria. Diabetologia.

[57] Nørgaard K., Feldt-Rasmussen B., Borch-Johnsen K., Saelan H., Deckert T. (1990). Prevalence of hypertension in type 1 (insulin-dependent) diabetes mellitus. Diabetologia.

[58] Puelles V.G., Zimanyi M.A., Samuel T., Hughson M.D., Douglas-Denton R.N., Bertram J.F., Armitage J.A. (2012). Estimating individual glomerular volume in the human kidney: Clinical perspectives. Nephrol. Dial. Transplant.

[59] Samuel T., Hoy W.E., Douglas-Denton R., Hughson M.D., Bertram J.F. (2005). Determinants of glomerular volume in different cortical zones of the human kidney. J. Am. Soc. Nephrol..

[60] Tsuboi N., Utsunomiya Y., Koike K., Kanzaki G., Hirano K., Okonogi H., Miyazaki Y., Ogura M., Joh K., Kawamura T. (2013). Factors related to the glomerular size in renal biopsies of chronic kidney disease patients. Clin. Nephrol..

[61] Tsuboi N., Okabayashi Y., Shimizu A., Yokoo T. (2017). The Renal Pathology of Obesity. Kidney Int. Rep..

[62] Hughson M.D., Hoy W.E., Douglas-Denton R.N., Zimanyi M.A., Bertram J.F. (2011). Towards a definition of glomerulomegaly: Clinical-pathological and methodological considerations. Nephrol. Dial. Transplant.

[63] Viggiano D., De Santo N.G., Amruthraj N.J., Capolongo G., Capasso G., Anastasio P. (2019). Renal response to an oral protein load in patients with central diabetes insipidus before and after treatment with vasopressin. J. Nephrol..

[64] Anastasio P., Viggiano D., Zacchia M., Altobelli C., Capasso G., Gaspare De Santo N., De Santo N. (2017). Delay in Renal Hemodynamic Response to a Meat Meal in Severe Obesity. Nephron.

[65] Chen J.-K., Nagai K., Chen J., Plieth D., Hino M., Xu J., Sha F., Ikizler T.A., Quarles C.C., Threadgill D.W. (2015). Phosphatidylinositol 3-kinase signaling determines kidney size. J. Clin. Investig..

[66] Kimmelstiel P., Wilson C. (1936). Intercapillary Lesions in the Glomeruli of the Kidney. Am. J. Pathol..

[67] Schwartz M.M., Lewis E.J., Leonard-Martin T., Lewis J.B., Batlle D. (1998). Renal pathology patterns in type II diabetes mellitus: Relationship with retinopathy. The Collaborative Study Group. Nephrol. Dial. Transplant.

[68] Najafian B., Alpers C.E., Fogo A.B. (2011). Pathology of human diabetic nephropathy. Contrib. Nephrol..

[69] Veron D., Bertuccio C.A., Marlier A., Reidy K., Garcia A.M., Jimenez J., Velazquez H., Kashgarian M., Moeckel G.W., Tufro A. (2011). Podocyte vascular endothelial growth factor (Vegf_164_) overexpression causes severe nodular glomerulosclerosis in a mouse model of type 1 diabetes. Diabetologia.

[70] Yuen D.A., Stead B.E., Zhang Y., White K.E., Kabir M.G., Thai K., Advani S.L., Connelly K.A., Takano T., Zhu L. (2012). eNOS deficiency predisposes podocytes to injury in diabetes. J. Am. Soc. Nephrol..

[71] Steffensen L.B., Iversen X.E.S., Hansen R.S., Jensen P.S., Thorsen A.-S.F., Lindholt J.S., Riber L.P.S., Beck H.C., Rasmussen L.M. (2021). Basement membrane proteins in various arterial beds from individuals with and without type 2 diabetes mellitus: A proteome study. Cardiovasc. Diabetol..

[72] Pozzi A., Yurchenco P.D., Iozzo R.V. (2017). The nature and biology of basement membranes. Matrix Biol..

[73] Zhou Y., Chang D.-Y., Li J., Shan Y., Huang X.-Y., Zhang F., Luo Q., Xiong Z.-Y., Zhao M.-H., Hou S. (2022). The role of Kimmelstiel-Wilson nodule in the kidney outcome in patients with diabetic kidney disease: A two-center retrospective cohort study. Diabetes Res. Clin. Pract..

[74] Preil S.A.R., Kristensen L.P., Beck H.C., Jensen P.S., Nielsen P.S., Steiniche T., Bjørling-Poulsen M., Larsen M.R., Hansen M.L., Rasmussen L.M. (2015). Quantitative Proteome Analysis Reveals Increased Content of Basement Membrane Proteins in Arteries From Patients With Type 2 Diabetes Mellitus and Lower Levels Among Metformin Users. Circ. Cardiovasc. Genet..

[75] Nakatani S., Wei M., Ishimura E., Kakehashi A., Mori K., Nishizawa Y., Inaba M., Wanibuchi H. (2012). Proteome analysis of laser microdissected glomeruli from formalin-fixed paraffin-embedded kidneys of autopsies of diabetic patients: Nephronectin is associated with the development of diabetic glomerulosclerosis. Nephrol. Dial. Transplant..

[76] Zhao L., Liu F., Li L., Zhang J., Wang T., Zhang R., Zhang W., Yang X., Zeng X., Wang Y. (2021). Solidified glomerulosclerosis, identified using single glomerular proteomics, predicts end-stage renal disease in Chinese patients with type 2 diabetes. Sci. Rep..

[77] Fazakerley D.J., Krycer J.R., Kearney A.L., Hocking S.L., James D.E. (2019). Muscle and adipose tissue insulin resistance: Malady without mechanism?. J. Lipid Res..

[78] Iovino S., Oriente F., Botta G., Cabaro S., Iovane V., Paciello O., Viggiano D., Perruolo G., Formisano P., Beguinot F. (2012). PED/PEA-15 induces autophagy and mediates TGF-beta1 effect on muscle cell differentiation. Cell Death Differ..

[79] Vigliotta G., Miele C., Santopietro S., Portella G., Perfetti A., Maitan M.A., Cassese A., Oriente F., Trencia A., Fiory F. (2004). Overexpression of the ped/pea-15 gene causes diabetes by impairing glucose-stimulated insulin secretion in addition to insulin action. Mol. Cell. Biol..

[80] Perruolo G., Viggiano D., Fiory F., Cassese A., Nigro C., Liotti A., Miele C., Beguinot F., Formisano P. (2016). Parkinson-like phenotype in insulin-resistant PED/PEA-15 transgenic mice. Sci. Rep..

[81] Ricci S., Viggiano D., Cimmino I., Perruolo G., Cabaro S., Liotti A., Fiory F., Spinelli R., Di Carlo A., Beguinot F. (2018). Prep1 Deficiency Affects Olfactory Perception and Feeding Behavior by Impairing BDNF-TrkB Mediated Neurotrophic Signaling. Mol. Neurobiol..

[82] Quiñones-Galvan A., Ferrannini E. (1997). Renal effects of insulin in man. J. Nephrol..

[83] Viggiano D., Gigliotti G., Vallone G., Giammarino A., Nigro M., Capasso G. (2018). Urate-Lowering Agents in Asymptomatic Hyperuricemia: Role of Urine Sediment Analysis and Musculoskeletal Ultrasound. Kidney Blood Press. Res..

[84] Nigro M., Viggiano D., Ragone V., Trabace T., di Palma A., Rossini M., Capasso G., Gesualdo L., Gigliotti G. (2018). A cross-sectional study on the relationship between hematological data and quantitative morphological indices from kidney biopsies in different glomerular diseases. BMC Nephrol..

[85] Safai N., Suvitaival T., Ali A., Spégel P., Al-Majdoub M., Carstensen B., Vestergaard H., Ridderstråle M., CIMT Trial Group (2018). Effect of metformin on plasma metabolite profile in the Copenhagen Insulin and Metformin Therapy (CIMT) trial. Diabet. Med..

[86] Lanza I.R., Zhang S., Ward L.E., Karakelides H., Raftery D., Nair K.S. (2010). Quantitative Metabolomics by 1H-NMR and LC-MS/MS Confirms Altered Metabolic Pathways in Diabetes. PLoS ONE.

[87] Skovsø S., Damgaard J., Fels J.J., Olsen G.S., Wolf X.A., Rolin B., Holst J.J. (2015). Effects of insulin therapy on weight gain and fat distribution in the HF/HS-STZ rat model of type 2 diabetes. Int. J. Obes..

[88] Takei I., Takayama S., Yamauchi A., Nakamoto S., Kitamura Y., Katsukawa F., Yamazaki H., Saruta T., Inoue S. (1998). Effect of insulin therapy on body fat distribution in NIDDM patients with secondary sulfonylurea failure: A preliminary report. Eur. J. Clin. Nutr..

[89] Apovian C.M., Okemah J., O’Neil P.M. (2019). Body Weight Considerations in the Management of Type 2 Diabetes. Adv. Ther..

[90] Hill D.J., Milner R.D.G. (1985). Insulin as a Growth Factor. Pediatr. Res..

[91] Monaco S., Illario M., Rusciano M.R., Gragnaniello G., Di Spigna G., Leggiero E., Pastore L., Fenzi G., Rossi G., Vitale M. (2009). Insulin stimulates fibroblast proliferation through calcium-calmodulin-dependent kinase II. Cell Cycle.

[92] Adel F.W., Zheng Y., Wan S.-H., Greason C., Pan S., Ameenuddin S., Chen H.H. (2022). Insulin Therapy is Associated with Increased Myocardial Interstitial Fibrosis and Cardiomyocyte Apoptosis in a Rodent Model of Experimental Diabetes. Front. Physiol..

[93] Lu C.-C., Chu P.-Y., Hsia S.-M., Wu C.-H., Tung Y.-T., Yen G.-C. (2017). Insulin induction instigates cell proliferation and metastasis in human colorectal cancer cells. Int. J. Oncol..

[94] Escudero C.A., Herlitz K., Troncoso F., Guevara K., Acurio J., Aguayo C., Godoy A.S., González M. (2017). Pro-angiogenic Role of Insulin: From Physiology to Pathology. Front. Physiol..

[95] Black E. (2003). Decrease of Collagen Deposition in Wound Repair in Type 1 Diabetes Independent of Glycemic Control. Arch. Surg..

[96] Kuo J.Z., Guo X., Klein R., Klein B.E., Weinreb R.N., Genter P., Hsiao F.-C., Goodarzi M.O., Rotter J.I., Chen Y.-D.I. (2014). Association of fasting insulin and C peptide with diabetic retinopathy in Latinos with type 2 diabetes. BMJ Open Diabetes Res. Care.

[97] Swinnen S.G., Hoekstra J.B., DeVries J.H. (2009). Insulin Therapy for Type 2 Diabetes. Diabetes Care.

[98] Marx N., Walcher D. (2008). C-Peptide and Atherogenesis: C-Peptide as a Mediator of Lesion Development in Patients with Type 2 Diabetes Mellitus?. Exp. Diabetes Res..

[99] Ahmadirad H., Teymoori F., Mokhtari E., Jahromi M.K., Norouzzadeh M., Tavakkoli S., Shahrokhtabar T., Farhadnejad H., Mirmiran P. (2023). Serum C-peptide level and the risk of cardiovascular diseases mortality and all-cause mortality: A meta-analysis and systematic review. Front. Cardiovasc. Med..

[100] Foo K., Sekhri N., Deaner A., Knight C., Suliman A., Ranjadayalan K., Timmis A.D. (2003). Effect of diabetes on serum potassium concentrations in acute coronary syndromes. Heart.

[101] Johansson B.L., Borg K., Fernqvist-Forbes E., Kernell A., Odergren T., Wahren J. (2000). Beneficial effects of C-peptide on incipient nephropathy and neuropathy in patients with Type 1 diabetes mellitus. Diabet. Med..

[102] Johansson B.L., Kernell A., Sjöberg S., Wahren J. (1993). Influence of combined C-peptide and insulin administration on renal function and metabolic control in diabetes type 1. J. Clin. Endocrinol. Metab..

[103] Johansson B.L., Sjöberg S., Wahren J. (1992). The influence of human C-peptide on renal function and glucose utilization in type 1 (insulin-dependent) diabetic patients. Diabetologia.

[104] Samnegård B., Jacobson S.H., Jaremko G., Johansson B.-L., Sjöquist M. (2001). Effects of C-peptide on glomerular and renal size and renal function in diabetic rats. Kidney Int..

[105] Lane P.W., Dickie M.M. (1958). The Effect of Restricted Food Intake on the Life Span of Genetically Obese Mice. J. Nutr..

[106] Roy S., Sala R., Cagliero E., Lorenzi M. (1990). Overexpression of fibronectin induced by diabetes or high glucose: Phenomenon with a memory. Proc. Natl. Acad. Sci. USA.

[107] Manazir S., Durrani H.M., Jawed F., Iqbal Naviwala H. (2021). Concurrent Presentation of Diabetic Nephropathy and Type 1 Diabetes Mellitus in a Pediatric Patient. Cureus.

[108] Lalayiannis A.D., Thompson C., Malcomson R., Milford D.V. (2016). Nodular glomerulosclerosis in a patient with cystic fibrosis, but not diabetes mellitus: A paediatric case. Respir. Med. Case Rep..

[109] Finne P. (2005). Incidence of End-stage Renal Disease in Patients with Type 1 Diabetes. JAMA.

[110] Wu J., Zhang R., Torreggiani M., Ting A., Xiong H., Striker G.E., Vlassara H., Zheng F. (2010). Induction of diabetes in aged C57B6 mice results in severe nephropathy: An association with oxidative stress, endoplasmic reticulum stress, and inflammation. Am. J. Pathol..

